# Modulation of Cyclic AMP Levels in Fallopian Tube Cells by Natural and Environmental Estrogens

**DOI:** 10.3390/cells10051250

**Published:** 2021-05-19

**Authors:** Marinella Rosselli, Barbara P. S. Cometti, Brigitte Leeners, Marta Ewa Szutkowska, Edwin K. Jackson, Raghvendra K. Dubey

**Affiliations:** 1 Department of Reproductive Endocrinology, University Hospital Zurich, Areal Wagi Schlieren, Wagistrasse 14, CH 8952 Schlieren, Switzerland; marinella.rosselli@usz.ch (M.R.); barbara.cometti@ibsa.ch (B.P.S.C.); brigitte.leeners@usz.ch (B.L.); Marta.Szutkowska@usz.ch (M.E.S.); 2 R&D Scientific Affairs, IBSA Institut Biochimique SA, Via del Piano 29, P.O. Box 266, CH 6915 Pambio-Noranco, Switzerland; 3 Department of Pharmacology & Chemical Biology, University of Pittsburgh, Pittsburgh, PA 15219, USA; edj@pitt.edu

**Keywords:** fertilization, endocrine disruptors, infertility, hormones, fallopian tube

## Abstract

Autocrine/paracrine factors generated in response to 17β-estradiol (E2) within the fallopian tube (FT) facilitate fertilization and early embryo development for implantation. Since cyclic AMP (cAMP) plays a key role in reproduction, regulation of its synthesis by E2 may be of biological/pathophysiological relevance. Herein, we investigated whether cAMP production in FT cells (FTCs) is regulated by E2 and environmental estrogens (EE’s; xenoestrogens and phytoestrogens). Under basal conditions, low levels of extracellular cAMP were detectable in bovine FTCs (epithelial cells and fibroblasts; 1:1 ratio). Treatment of FTCs with forskolin (AC; adenylyl cyclase activator), isoproterenol (β-adrenoceptor agonist) and IBMX (phosphodiesterase (PDE) inhibitor) dramatically (>10 fold) increased cAMP; whereas LRE1 (sAC; soluble AC inhibitor) and 2’,5’-dideoxyadenosine (DDA; transmembrane AC (tmAC)) inhibitor decreased cAMP. Comparable changes in basal and stimulated intracellular cAMP were also observed. Ro-20-1724 (PDE-IV inhibitor), but not milrinone (PDE-III inhibitor) nor mmIBMX (PDE-I inhibitor), augmented forskolin-stimulated cAMP levels, suggesting that PDE-IV dominates in FTCs. E2 increased cAMP levels and CREB phosphorylation in FTCs, and these effects were mimicked by EE’s (genistein, 4-hydroxy-2’,4’,6’-trichlorobiphenyl, 4-hydroxy-2’,4’,6’-dichlorobiphenyl). Moreover, the effects of E2 and EE were blocked by the tmAC inhibitor DDA, but not by the ERα/β antagonist ICI182780. Moreover, BAPTA-AM (intracellular-Ca^2+^ chelator) abrogated the effects of E2, but not genistein, on cAMP suggesting differential involvement of Ca^2+^. Treatment with non-permeable E2-BSA induced cAMP levels and CREB-phosphorylation; moreover, the stimulatory effects of E2 and EEs on cAMP were blocked by G15, a G protein-coupled estrogen receptor (GPER) antagonist. E2 and IBMX induced cAMP formation was inhibited by LRE1 and DDA suggesting involvement of both tmAC and sAC. Our results provide the first evidence that in FTCs, E2 and EE’s stimulate cAMP synthesis via GPER. Exposure of the FT to EE’s and PDE inhibitors may result in abnormal non-cyclic induction of cAMP levels which may induce deleterious effects on reproduction.

## 1. Introduction

An optimal autacoid microenvironment within the fallopian tube (FT) is necessary for fertilization and early embryo development and is, therefore, essential for reproduction [1]. For example, several autocrine-paracrine factors generated within the FT regulate cyclic contraction and relaxation of FT smooth muscle cells and ciliary movement of FT epithelial cells, processes that are required for transporting gametes and embryos [1]. As we recently described [2], FT cells produce a number of autacoids including nitric oxide, hydrogen sulphide, prostaglandins, thromboxane, adenosine, leukaemia inhibitory factor, inflammatory cytokines, endothelin, transforming growth factor-β and microRNAs. Furthermore, FT cells secrete extracellular vesicles [3] which transport signaling molecules to surrounding cells [4].

The autacoid microenvironment within the FT can influence the synthesis of intracellular signaling molecules, including cyclic AMP (cAMP), by FT cells. cAMP is a ubiquitous second messenger that plays an important role in reproduction [1,5,6]. Hormonal stimulation rapidly increases cAMP levels within FT cells by approximately five-fold, and this newly generated cAMP has diverse effects on cellular biology. For example, intracellular cAMP regulates cell growth and has been shown to both stimulate as well as inhibit cell growth [7,8]. Specifically, cAMP inhibits cell growth at high concentrations [8]. cAMP also relaxes smooth muscle cells [9].

Because cAMP exerts such a powerful influence on the biology of cells, intricate mechanisms have evolved to regulate cAMP levels. When activated by G-protein-coupled receptors, membrane-localized Gαs subunits are released from a trimeric complex (Gαβγ) and activate membrane-bound adenylyl cyclase (AC) which synthesizes cAMP from ATP. To avoid high and prolonged increases in intracellular cAMP, one or more isoforms of phosphodiesterases (PDEs) hydrolyze cAMP to adenosine 5′-monophosphate (5′-AMP). Of the 11 PDE families, PDE 4, PDE7 and PDE 8 selectively hydrolyze cAMP, whereas PDE 1 (Ca^2+^/calmodulin dependent), 2, 3, 10 and 11 possess dual specificity for cAMP and cGMP. PDEs 1, 2, 3 and 4 are not ubiquitous but are expressed in many tissues, whereas others are more restricted. In most cells, PDE3 and PDE4 provide the major portion of cAMP-hydrolyzing activity [10,11]. In addition to hydrolysis by PDEs, intracellular cAMP is removed from cells by efficient export to the extracellular space [12,13,14]. Because extracellular cAMP has direct and indirect autocrine/paracrine effects [9], egress of cAMP serves to broaden the signaling roles of cAMP.

cAMP has multiple effects in the reproductive system. For example, cAMP regulates sperm motility and sperm capacitation [6]. cAMP also influences the mammary gland, and cAMP levels in the mammary gland increase progressively during pregnancy, whereas at the time of parturition cAMP levels decrease precipitously [15]. cAMP regulates steroidogenesis by modulating aromatase activity in the ovary [16] and actively contributes to the cyclic contractility of the oviduct to facilitate fertilization and early embryo development and transit for implantation [17]. Apart from influencing muscle tone and motility, cAMP is a potent regulator of growth [7], and high concentrations of cAMP induce anti-mitogenic actions [7,8]. Within the ovary, cAMP plays a crucial role in oocyte maturation [18,19]. Spontaneous maturation of mouse oocytes does not occur in vitro when oocytes are cultured in the presence of either membrane permeable analogs of cAMP, inhibitors of cAMP hydrolysis [14], or adenylyl cyclase activators [20]. In mice lacking PDE3A, increases in oocyte cAMP levels are associated with abnormal oocyte maturation and female infertility [19]. This suggests that cAMP is involved in the maintenance of meiotic arrest at the dictyate stage, and a fall in intracellular cAMP levels could signal re-entry of oocyte into meiotic progression.

Interestingly, treatment of embryos with agents that increase cAMP levels, for example AC activators or PDE inhibitors, induces deleterious effects on embryo maturation suggesting that cAMP can influence embryo growth and fertility [21,22]. Drugs known to elevate cAMP by inhibiting PDEs have been shown to impair oviductal embryo transport, embryo development and uterine receptivity [23,24,25,26]. Whether these effects are a result of cAMP produced within the FT or due to direct actions on the embryo is unclear. Since extracellular cAMP is known to be biologically active [27], it is feasible that excessive cAMP produced within the FT may, in part, contribute to these deleterious actions. Since early embryo development occurs within the FT it is important to assess the impact of various PDEs on cAMP synthesis by FT cells. Since PDE exists in various isoforms, we investigated which PDE is present in FT cells and regulates cAMP levels.

There is an important connection between the ovarian hormone 17β-estradiol (E2) and cAMP. E2 is known to stimulate cAMP synthesis in reproductive system. This effect of E2 is receptor mediated and involves both genomic and non-genomic mechanisms [28]. Accumulating data suggest that environmental estrogens (EEs) induce deleterious effects on the reproductive system [29,30,31] and contribute to infertility. However, the mechanisms involved remain unclear. Bisphenol A, a EE used in plastics, has been shown to negatively affect early embryo development and metabolism [30]. Since EEs mimic the effects of E2 on multiple FT-derived factors [2], it is feasible that EEs also induce cAMP in FT cells and influence FT function. Indeed, unlike the physiological cyclical changes in E2, exposure to EEs may result in non-cyclical stimulation of cAMP, and this may induce deleterious effects on early embryo development and consequently on fertility. How E2 regulates cAMP synthesis in the FT cells and whether EE’s mimic the effects of E2 on cAMP synthesis and on the downstream signaling involving cAMP response element binding protein phosphorylation (pCREB) remain unknown.

Degradation of intracellular cAMP by PDEs is widely postulated to terminate cAMP signaling; however, increasing evidence suggests that significant amounts of cAMP are actively expelled and may have a direct or indirect pathophysiological role on surrounding cells. Indeed, a role for extracellular cAMP is well demonstrated within the cardiac, renal, cerebral cortex, gastrointestinal, skeletal muscle, and glucagon mediated endocrine actions [27,28,32,33,34,35,36]. Extracellular cAMP has also been shown to play an important role in sperm motility and in activating molecular signaling pathways associated with sperm capacitation [27]. The egress of cAMP in mammalian cells is regulated by multi-drug resistance proteins including MRP4 and MRP5, which are expressed within the reproductive tract [37]. Hence cAMP egress from FT cells is a feasible mechanism to influence local processes in an autocrine and paracrine fashion. Since early embryo development is influenced by the local oviduct environment, changes in extracellular cAMP levels may be of pathophysiological relevance. We have previously shown that oviduct cells efficiently metabolize extracellular cAMP to generate adenosine [38], which in turn can activate adenosine receptors and induce intracellular cAMP in surrounding cells and tissue. Based on these observations and our hypothesis that extracellular cAMP within the FT may influence embryo development, we decided to assay extracellular cAMP. Moreover, we also assessed whether changes in intracellular cAMP and CREB phosphorylation were compatible with changes in extracellular cAMP in FT cells.

Given the important roles of cAMP in the reproductive system in general and the FT more specifically, the aims of the present study were to investigate: (i) whether FT cells efficiently produce cAMP; (ii) the identity of PDE isoforms responsible for cAMP catabolism in FT cells; (iii) whether E2 can modulate cAMP production in FT cells; (iv) whether EEs can influence cAMP production in FT cells; (v) whether the effects of E2 and EEs on cAMP in FT cells are mediated via estrogen receptors (ERα, ERβ or G protein coupled estrogen receptor (GPER)); (vi) whether E2 and EEs trigger phosphorylation of cAMP response element binding protein (CREB) in FT cells; and (vii) which mechanisms are involved in E2 and EEs effects on cAMP synthesis and CREB phosphorylation in FT cells.

## 2. Materials and Methods

### 2.1. Mixed Cultures

FTs of young, cyclic, non-pregnant cows (Braunvieh; 20–26 months old; *n* = 24), were obtained from the local abattoir and FT cells (mixed population of epithelial cells and fibroblast, 1:1 ratio) were obtained and cultured according to our previously published method [2]. Briefly, FTs were placed immediately in ice-cold Ca^2+^- and Mg^2+^-deficient Hanks’ balanced salt solution (HBSS; Bioconcept/Amimed, Switzerland) containing 100 μg/mL streptomycin, 100 μg/mL penicillin and 0.025 μg/mL amphotericin B (Gibco, Life Technologies, Grand Island, NY, USA). The FTs were separated from surrounding tissue, washed several times with HBSS, and placed under sterile conditions in petri dishes containing HBSS. Subsequently, the FTs were cut open longitudinally and the inner lining of the lumen was gently scraped with a cell scraper. The scraped cells were collected and suspended in HBSS and washed by centrifugation at 500× *g*. The final pellet was suspended in Ham’s F10 (Sigma, Chemie, Buchs, Switzerland) supplemented with 10% fetal bovine serum (FBS), 100 μg/mL streptomycin, 100 μg/mL penicillin and 0.025 μg/mL amphotericin B (all from Gibco). FT cells were allowed to grow to sub-confluence. Confluent monolayers of FT cells were cultured for 6–8 days and used in first passage. The mixed cell cultures of FT epithelial cells and FT fibroblasts were characterized immunohistochemically as previously described [2] and as shown in Supplementary Figure S1. Monoclonal antibodies to epithelial cell cytokeratin, (anti-cytokeratin AE1/AE3; Dako Diagnostics AG, Zug, Switzerland) and antibodies against fibroblast vimentin (anti-vimentin VIM 3B4; Dako) were used to identify FT epithelial and fibroblasts in culture, respectively. Stained cells were visualized using peroxidase-anti-peroxidase staining (Dako).

### 2.2. Cyclic AMP Synthesis in Cultured Bovine FT Cells

Cultures of mixed FT cells were used to assess cAMP synthesis. Prior to experiments with mixed cultures preliminary experiments in cultured FT fibroblasts (>95% purity) and epithelial cells (>95% purity) were conducted to assess cAMP synthesis. In supernatants collected from FT epithelial cells and FT fibroblast comparable changes in cAMP levels were observed in response to PDE inhibitor IBMX, adenylyl cyclase stimulator forskolin and E2 plus IBMX (Supplementary Figure S2). To assess cAMP synthesis, bovine FT cells were cultured to sub-confluence in DMEM/F12 Ham’s medium containing 10% steroid free fetal bovine serum (SF-FBS). Subsequently monolayers of FT cells were washed with HBSS and incubated with DMEM/F12 Ham’s medium supplemented with 1% SF-FBS and containing or lacking adenylyl cyclase activators, forskolin (0.1–10 μM; Alexis Biochemicals) or isoproterenol (0.1–10 μM; Sigma). After 15 min of treatment, the conditioned medium was collected for cAMP analysis and the cells solubilized in 0.2% sodium dodecylsulfate (SDS) and the protein measured using BCA-protein assay kit (Socochim SA, Lausanne, Switzerland). To assess PDE activity, cultured bovine FT cells were treated with forskolin (0.5 µM) in presence and absence of various PDE inhibitors IBMX (0.1–100 μM; broad spectrum PDE inhibitor; Sigma), mmIBMX (100 μM; selective PDE-I inhibitor; Calbiochem), Ro 20-1724 (0.1–500 μM; selective PDE-IV inhibitor; Calbiochem) or milrinone (10 μM; selective PDE-III inhibitor; Sigma). Effects of higher concentrations (0-1mM) of IBMX and mmIBMX on forskolin-induced cAMP in FT cells were also tested to confirm their modulatory actions on cAMP levels.

To investigate whether the synthesis of cAMP is modulated by E2 or EEs, mixed cultures were incubated for 15 min with DMEM/Ham F12 supplemented with 1% charcoal-stripped FBS and containing or lacking different concentrations (0–200 ng/mL) of E2, genistein, trichlorobiphenyl (TCB), 4-hydroxy-2′,4′,6′-dichlorobiphenyl (4OH-DCB), or 4-hydroxy-2′,4′,6′-trichlorobiphenyl (4-OH-TCB). To assess whether E2 and EEs induce their effects via estrogen receptors (ERs) α or β, FT cells were treated with E2 or EEs in presence of ICI182780 (1 μM; Tocris), an ERαβ antagonist. To investigate the role of membrane ERs, cells were treated with BSA tagged E2 (E2-BSA; normalized to achieve E2 concentrations of 2 ng/mL; Sigma) which is membrane impermeable. Moreover, to investigate the role of the membrane G-protein-coupled ER, namely GPER, the effects of G1 (25 nM, GPER agonist, TOCRIS), E2 and EEs on cAMP levels were measured in the presence and absence of G15 (250 nM; Tocris), a GPER antagonist. To assess the role of adenylyl cyclase, cells were treated with E2 or EEs in the presence of 2′5′-dideoxyadenosine (DDA; 10 µM; Sigma), a transmembrane adenylyl cyclase (tmAC) inhibitor or with LRE1 (50 µM), a soluble AC inhibitor. For each experiment, the conditioned medium was collected and levels of extracellular cAMP analyzed. As described below, intracellular cAMP in was also measured in some experiments to confirm that extracellular cAMP levels reflect changes in intracellular levels.

To assess cAMP formation in freshly isolated FT cells, cells were washed 3 times with HBSS. Aliquots of cell suspensions (100 μL, corresponding to 0.3 mg of protein) were added to 400 μL solution containing the various treatments and incubated for 15 min at 37 °C in presence or absence of the desired treatment. cAMP levels were measured in 100 ul aliquots using the Biotrack EIA system as described below.

To compare the modulatory actions of the various treatments on changes in intracellular and extracellular cAMP levels, monolayers were washed with HBSS and incubated with 800 μL of DMEM/F12 Ham’s medium supplemented with 1% SF-FBS and containing or lacking the various treatments. After the desired incubation the medium was collected, to assess extracellular cAMP, whereas the monolayers washed once with ice old HBSS and subsequently, 800 μL of DMEM/F12 Ham’s medium supplemented with 1% SF-FBS added. Plates were immediately frozen (to rupture the cells) and subsequently thawed, and the cells scraped in Eppendorf tubes centrifuged at the supernatant collected for intracellular cAMP analysis. The pellet was dissolved in SDS (0.2%) and protein measured with BCA kit, as described above.

### 2.3. Cyclic AMP Assay

It is well known that in metazoan cells stimulation of adenylyl cyclase increases both intracellular and extracellular cAMP levels. This is because intracellular cAMP produced by adenylyl cyclase is rapidly transported to the extracellular compartment. Consequently, extracellular cAMP is a reliable index of changes in cellular cAMP production; and in the present study we largely measured cAMP levels in the conditioned medium as an index of cellular cAMP production. We compared and confirmed the comparable changes in intracellular and extracellular cAMP in FT cells, in response to some important treatments (see results). There are two advantages for using this approach. First the samples are cleaner so that the assay is more reliable. Second, extracellular cAMP not only reflects intracellular cAMP signaling, but also indicates the potential for extracellular signaling via conversion of cAMP to adenosine. Hence, in this investigation the changes in cAMP were largely measured in the conditioned medium and clearly described as extracellular (Ex).

Aliquots (100 μL) of conditioned medium were used to analyze the presence of cAMP using an ELISA Kit (Biotrak, cAMP enzyme-immunoassay (EIA) system, Amersham Pharmacia Biotech, UK). Assays were performed according to the manufacturer’s specification. Briefly, 100 μL of each sample was added to a microtiter plate coated with donkey anti-rabbit IgG. Then, 100 μL of antiserum containing a rabbit anti-cAMP antibody was added and the plate was incubated at 4 °C for exactly 2 h. Subsequently, 50 μL of cAMP- horseradish peroxidase conjugate was added and the plate incubated at 4 °C for 60 min. After this, all wells were washed several times and 150 μL of enzyme substrate containing 3,3′,5,5′-tetramethylbenzidine (TMB)/hydrogen peroxide, in 20% (*v*/*v*) dimethylformamide, was added. The plate was then incubated at room temperature for 30 min. The reaction was stopped using 100 μL 1.0 M sulfuric acid, and the optical density determined in a plate reader (Bio-Rad microplate reader) at 450 nm. The concentrations of cAMP were estimated using a standard curve run under identical conditions. The cAMP levels reported as pmol/mL/mg protein.

### 2.4. Measurement of PDE Activity (Enzymatic Assay) on Disrupted FT Cells

To assess whether E2 can increase cAMP by modulating PDE activity in FT cells, confluent monolayers were washed thrice with HBSS and subsequently sonicated for three 30 s periods. Protein concentration of disrupted cells was measured and the solution diluted to achieve a final concentration of 0.5 mg protein/mL. In total, 500 μL of disrupted cells was added to 500 μL HBSS containing 30 nM cAMP with or without various treatments (E2 at 0.2–200 ng/mL or PDE inhibitor) and incubated for 4 h. Aliquots of 100 µL were collected and cAMP levels were measured using the Biotrack EIA system as described above. The values are reported as pmol/mL/mg protein.

### 2.5. Modulation of CREB Phosphorylation

Western blotting (Bio-Rad protean system) was employed to assess the impact of E2 and EEs on CREB phosphorylation. To identify optimal conditions for CREB phosphorylation, sub-confluent cells were treated with 20 ng/mL of E2 for different times (0–30 min) and then phosphorylated CREB was assayed. Since optimal phosphorylation was evident between 15 and 30 min, FT cells grown to sub-confluence were treated at 37 °C for 20 min with forskolin (50 µM) and either E2 (2, 20 ng/mL) or 20 ng/mL of genistein or 4-OH-TCB. To assess the role of membrane ERs, FT cells were treated with membrane impermeable BSA tagged E2 with the final concentration of 20 ng/mL E2 (after correction for BSA molecular weight). To assess the role of ERs αβ, GPR30, adenylyl cyclase, intracellular calcium, sAC and tmAC, the above treatments were performed in presence or absence of ICI182780 (1 µM), G15 (250 nM), DDA (100 µM), BAPTA-AM (1 µM), or LRE1 (50 µM), respectively. The cells were washed and solubilized in lysis buffer (Cell Signal) and protein concentration measured with BCA protein assay kit. Aliquots containing 20 µg protein were loaded and p-CREB quantified by western blotting and using anti-phospho-CREB (rabbit immunoaffinity purified IgG, Upstate Biotechnology, NY, USA) diluted to 1:1000 or with anti-CREB (rabbit polyclonal IgG, Upstate Biotechnology) used at 1 μg/mL. After washing the bands were determined using Super signal west dura luminol substrate (Pierce) and Hyperfilm ECL films (Amersham). The optical density of the p-CREB was normalized with CREB and the ratio (arbitrary units) used to assess differences. The units for changes in OD are presented as pCREB to CREB ratio or percent of control.

### 2.6. Receptor Expression Studies

The expression of membrane estrogen receptor GPR30 in FT cells was assessed using western blotting as well as by immunostaining. Briefly, FT cell lysates were probed with antibodies for GPR30 (GPR30 (N-15)-R; sc-48525-R, rabbit polyclonal antibody raised against a peptide mapping N-terminus of human GPR30; Santa Cruz Biotechnology; dilution 1:1000; recognizes GPR30 at ≈50 kDa). A compatible secondary antibody was used for band visualization (ImmunoPure goat anti-rabbit IgG, peroxidase conjugated, Pierce, IL, USA). The secondary antibody was diluted to 1:25,000 in 1% milk, PBS, Tween 20 0.2% buffer, and incubated for 1 h at room temperature. For detection of GPR30 in FT cells, stained cells were visualized using peroxidase, anti-peroxidase staining.

### 2.7. Protein Estimation

To determine protein concentrations, cells remaining after removal of cell culture supernatant were solubilized in 0.2% SDS and the protein assayed with the BCA-protein assay kit (Sochochim, Lausanne, Switzerland) using bovine serum albumin as a standard.

*Statistical Analysis:* Data are presented as a mean and SEM of experiments conducted in triplicates (*n* = 3). Statistical analysis was performed using Statview and significance was calculated by ANOVA and Fisher’s least significant difference test. A value of *p* < 0.05 was considered statistically significant.

## 3. Results

**Figure 1:**
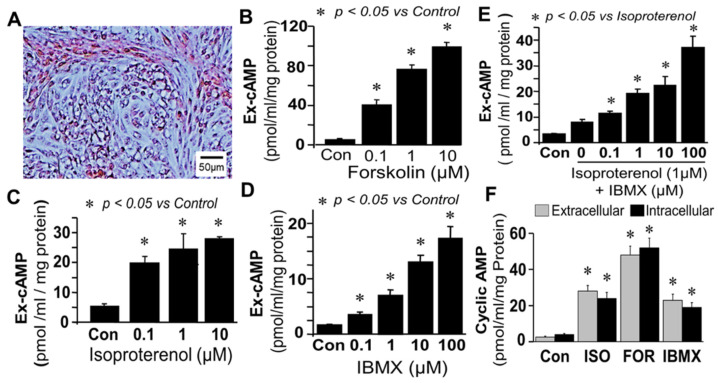
Panel (**A**) depicts a representative photomicrograph (40x magnification) of FT cells (mixed cultures of epithelial cells and fibroblasts 1:1 ratio). Bar graphs show the concentration-dependent stimulatory effects of treatments on extracellular (Ex) cAMP levels in confluent monolayer of FT cells after 15 min stimulation with: forskolin (0.1, 1, 10 μM), Panel (**B**); isoproterenol (0.1, 1, 10 μM), Panel (**C**); IBMX (0.1, 1, 10, 100 µM), panel (**D**); and IBMX (0.1–100 µM) plus isoproterenol (1 µM), Panel (**E**). Bar graph in Panel (**F**) shows changes in intracellular and extracellular cAMP levels in response to 1µM forskolin (FOR), 10 µM isoproterenol (ISO) or 1 µM IBMX. Data (mean and SEM) in bar graphs represent the mean of three different experiments (*n* = 3), with each experiment conducted in triplicate. Values were normalized to total protein concentration and the cAMP levels are expressed as pmol/mL/mg protein. * *p* < 0.05 vs. control.

Synthesis and regulation of cAMP in bovine FT cells: Treatment of monolayers of FT cells (containing epithelial cells and fibroblast in 1:1 ratio; Figure 1A) with forskolin (0.1–10 µM; adenylyl cyclase activator) increased extracellular cAMP levels in a concentration-dependent manner (Figure 1B). Stimulatory effects of greater than 10-fold (*p* < 0.05 vs. control) were observed at concentrations of 1 µM, and increases of 1851.3 ± 90 percent of control were observed with 10 µM of forskolin. Similar to forskolin, treatment of FT cells with isoproterenol (0.1–10 µM; β-adrenoceptor agonist) increased extracellular cAMP levels in a concentration-dependent manner (Figure 1C); however, compared to forskolin the magnitude of its stimulatory effects were lower. Significant levels of cAMP were also observed in cells treated with IBMX alone (Figure 1D). Treatment with isoproterenol (1 µM) in presence of increasing concentrations (0.1–100 µM) of IBMX (PDE inhibitor) increased extracellular cAMP levels in a concentration-dependent manner (*p* < 0.05 vs. isoproterenol alone; Figure 1E) with a maximal increase of ≈10 fold (*p* < 0.05 vs. control) at 100 µM of IBMX, suggesting that there was basal synthesis of cAMP which was rapidly metabolized by PDEs. Changes in extracellular cAMP in response to isoproterenol, forskolin and IBMX were comparable to intracellular (Figure 1F) suggesting that extracellular cAMP is a reliable marker for changes within FT cells.

**Figure 2:**
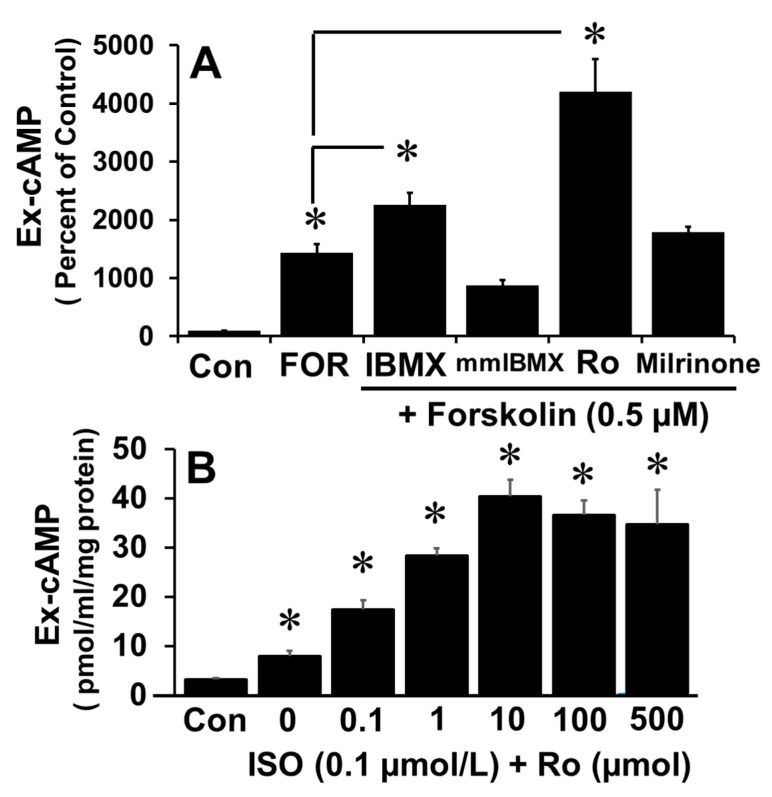
Panel (**A**) depicts bar graph showing the effects of different PDE isoform inhibitors on forskolin-induced increases in extracellular (Ex) cAMP levels after 15 min stimulation with: IBMX (100 μM); mmIBMX (100 μM), Ro20-1724 (100 μM); and milrinone (10 μM) on cAMP production in response to forskolin (0.5 μM) by confluent monolayer of FT cells. Panel (**B**) depicts the concentration-dependent modulatory actions of the PDE-IV inhibitor Ro20-1724 (Ro; 0.1–500 µM) on isoproterenol (0.1 µM) induced increases in cAMP levels. Data (mean and SEM) represent the mean of three different experiments (*n* = 3) in triplicate. Values were normalized to total protein concentration and the cAMP level is expressed as pmol/mL/mg protein or as percent of control (* *p* < 0.05 vs. control).

The fact that IBMX increases cAMP levels suggests that cAMP levels are regulated by PDEs. PDE isoforms including PDE-I, PDE-III and PDE-IV play a role in oocyte maturation; moreover, inhibitors of PDE isoforms influence early embryo development, although the relative role of the different isoforms varies. PDE-I is Ca^2+^/calmodulin regulated and has dual specificity for cAMP and cGMP; PDE-III is not ubiquitous and hydrolyzes both cAMP and cGMP, and regulates oocyte and embryo development [18,19,20,21] and PDE-IV is ubiquitously expressed and selectively hydrolyses cAMP [11]. To determine which isoform is dominant in FT cells in this regard, the increase in cAMP levels in response to forskolin was measured in the presence of 100 µM of mmIBMX (PDE-I inhibitor), milrinone (PDE-III inhibitor), Ro 20-1724 (Ro; PDE-IV inhibitor) or IBMX (non-specific PDE inhibitor). As shown in Figure 2A, Ro and IBMX, but not mmIBMX nor milrinone, augmented the effects of forskolin on cAMP levels. Furthermore, Ro augmented, in a concentration-dependent manner, the isoproterenol-induced increase in cAMP levels (*p* < 0.05 vs. ISO alone; Figure 2B). Concentration-response (0–1 mM) effects of IBMX and mmIBMX showed that higher concentrations (>100 uM) of IBMX, but not mmIBMX, further induced extracellular cAMP (Supplementary Figure S3), hence the reduced stimulatory effects compared to Ro20-1724 are related to the suboptimal IBMX concentration used. Moreover, lack of modulatory effects by mmIBMX on forskolin-induced cAMP at higher concentrations suggest that PDE-I may not regulate cAMP in FT cells.

**Figure 3:**
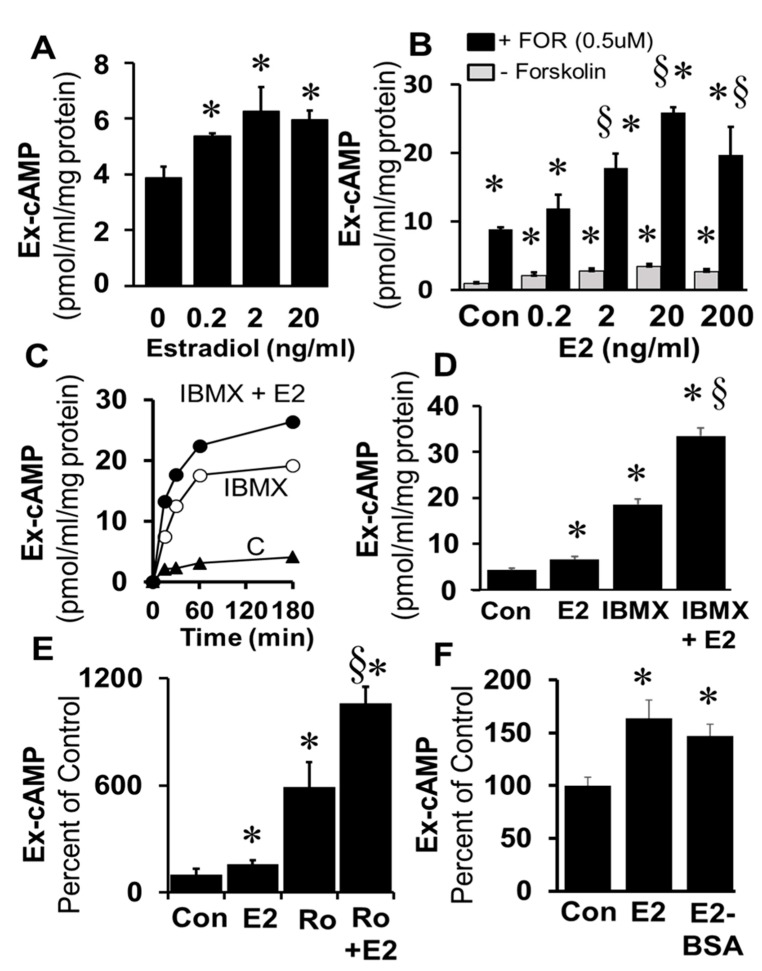
Panel (**A**) shows the concentration-dependent effects of 17β-estradiol (E2; 0.2, 2, 20 ng/mL) on extracellular (Ex) cAMP levels in freshly prepared FT cells treated for 15 min. Panel (**B**) depicts the stimulatory effects of E2 (0.2, 2, 20, 200 ng/mL) in confluent monolayers of FT cells in the presence of the adenylyl cyclase stimulator forskolin (0.5 µM). Panel (**C**) shows the time-dependent increases in cAMP levels in FT cells treated for 0–180 min with IBMX (1 µM) or IBMX (1 µM) plus E2 (20 ng/mL) compared with untreated control (**C**) cells. Panel (**D**) depicts the stimulatory effects of E2 (20 ng/mL) on cAMP levels in FT cells in presence of IBMX (1 µM) after 180 min. Panel (**E**) shows the stimulatory effects of E2 (20 ng/mL) on cAMP levels in FT cells in presence of Ro20-1724 (1 µM) after 180 min. Panel (**F**) shows the stimulatory effects of E2 and its membrane impermeable analog (E2-BSA) on cAMP levels in cultured FT cells treated for 15 min. Data (mean ± SEM) represent the mean of three different experiments (*n* = 3) in triplicate. Values were normalized to total protein concentration and the cAMP level is expressed as pmol/mL/mg protein or as percent of control (* *p* < 0.05 vs. control; ^§^ *p* < 0.05 vs. Ro or IBMX or Forskolin alone).

Treatment of freshly prepared and cultured FT cells (1× passage) with E2 (0.2–20 ng/mL) significantly increased cAMP levels (Figure 3A). The stimulatory effects of E2 on cAMP levels were significantly enhanced in the presence of the adenylyl cyclase activator forskolin (0.5 µM) and these effects were concentration-dependent (Figure 3B). Time-dependent (0–180 min; Figure 3C) stimulatory effects of E2 on levels of cAMP were also observed in FT cells treated simultaneously with IBMX (1 µM; *p* < 0.05 vs. IBMX alone). Furthermore, treatment for 180 min with E2 increased cAMP levels; and this response to E2 was greater in the presence of IBMX (Figure 3D) or Ro20-1724 (Figure 3E). The fact that the stimulatory effects of E2 on cAMP generation were rapid suggests the involvement of a non-genomic mechanism. To assess whether these effects were mediated via membrane ERs, the effects of E2-BSA (a non-permeable ER agonist) were compared with E2. Indeed, treatment with E2-BSA induced cAMP in FT cells and the stimulatory effects of E2 and E2-BSA did not differ (Figure 3F).

**Figure 4:**
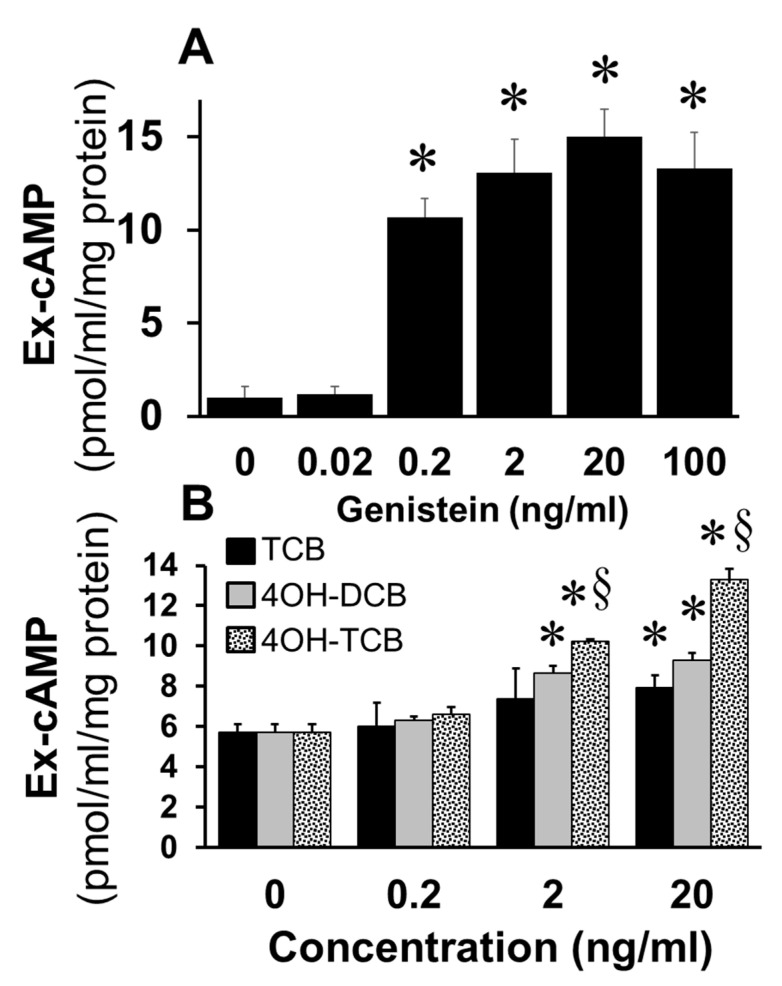
Panel (**A**) shows the concentration-dependent effects of genistein on extracellular (Ex) cAMP production by cultured FT cells treated for 15 min. Panel (**B**) depicts the concentration-dependent effects of TCB, 4-OH-TCB, and 4-OH-DCB on extracellular cAMP production by cultured FT cells, treated for 15 min. Data (mean ± SEM) represent the mean of three different experiments (*n* = 3) in triplicate, and values were normalized to total protein concentration. The levels of cAMP are expressed as pmol/mL/mg protein. * *p* < 0.05 vs. control and ^§^ *p* < 0.05 versus TCB.

To assess whether EEs mimic the effects of E2 on cAMP synthesis in FT cells, we studied the effects of genistein, a phytoestrogen, and biphenyls (TCB; 4OH-DCB; 4OH-TCB). As shown in Figure 4A, treatment of FT cells with genistein at concentrations of 0.2 to 100 ng/mL significantly increased cAMP levels (*p* < 0.05 vs. untreated control). Similarly, treatment with TCB, 4OH-DCB and 4OH-TCB increased cAMP levels and in the following order of potency 4OH-TCB > 4OH-DCB > TCB (Figure 4B).

**Figure 5:**
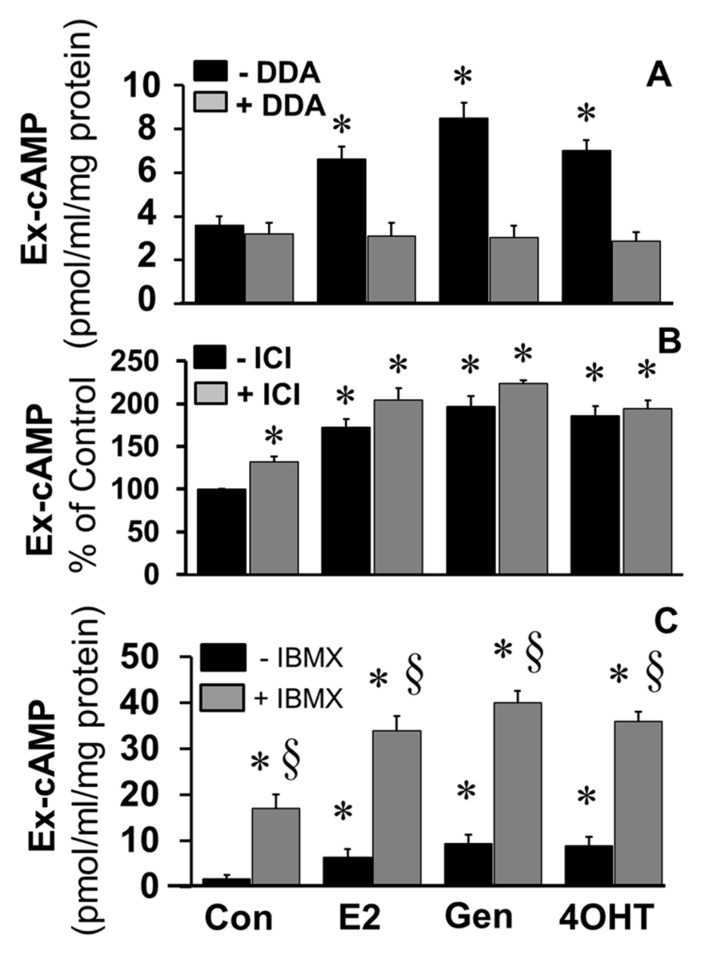
Bar graphs depicting the effects of 17β-estradiol (E2; 20 ng/mL), genistein (Gen; 20 ng/mL) and 4OH-TCB (4OHT; 20 ng/mL) on extracellular (Ex) cAMP production by FTCs stimulated for 15 min in absence or presence of: Panel (**A**) the transmembrane adenylyl cyclase inhibitor 2′5′-dideoxyadenosine (DDA; 10 µM); Panel (**B**) the estrogen receptor-αβ antagonist, ICI182780 (ICI; 1 µM); and Panel (**C**) the PDE inhibitor IBMX (1 µM). Data (mean ± SEM) represent the mean of three different experiments (*n* = 3) in triplicates. Values were normalized to total protein concentration and the cAMP levels are expressed as pmol/mL/mg protein or percent of control. * *p* < 0.05 vs. respective control; ^§^ *p* < 0.05 vs. treatment in absence of IBMX).

To elucidate the role of adenylyl cyclase in mediating the stimulatory effects of E2 and EEs, the effects of DDA, a tmAC inhibitor, on the stimulatory effects of E2, genistein, and 4OH-TCB were investigated. As shown in Figure 5A, the stimulatory effects E2, genistein, and 4OH-TCB on cAMP levels were abrogated in FT cells treated with DDA. To assess the role of ERs in mediating the effects of E2 and EEs, the stimulatory actions of E2, genistein, and 4OH-TCB on cAMP levels were assessed in the presence of ICI182780, an ERα/β antagonist. Treatment with ICI182780 resulted in a small but significant increase in cAMP levels; moreover, ICI182780 failed to block the stimulatory actions of E2, genistein and 4OH-TCB (Figure 5B), suggesting that the stimulatory effects of these compounds were independent of ERα/β. Since ICI182780 has been shown to act as an agonist for GPER [39], a membrane ER, it is feasible that the observed stimulatory effects of ICI182780 were via activation of a membrane ER. As shown in Figure 5C, the ability of E2, genistein and 4OH-TCB to increase levels of cAMP was increased by co-treatment with IBMX.

To assess whether changes in extracellular cAMP in response to E2 and EEs reflect parallel changes within the intracellular compartment, we simultaneously measured intracellular and extracellular cAMP. As shown in Supplementary Figure S4 comparable changes in intracellular and extracellular cAMP levels were observed in response to E2 and EEs (Gen and 4OHT).

## 4. Effects of E2 and EEs on CREB Phosphorylation

**Figure 6:**
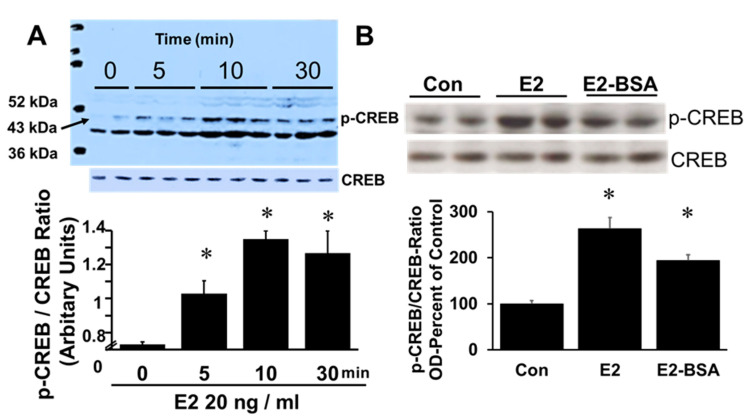
Panel (**A**) is a representative western blot showing the time-dependent (5, 10 and 30 min) phosphorylation of CREB (43 kDa protein) in response to 17β-estradiol (E2; 20 ng/mL) and Panel (**B**) shows a blot with CREB phosphorylation after 30 min, in response to E2 (20 ng/mL) and BSA tagged E2 (E2-BSA; 20 ng/mL). Bar graph depicts densitometric analysis for changes in CREB phosphorylation and presented in arbitrary units following normalization with non-phosphorylated CREB. Data (mean ± SEM) are from 3 separate experiments each in duplicates. Results were normalized by the internal standard, CREB. * *p* < 0.05 vs. 0 min or untreated control.

Treatment of FT cells with E2 increased immuno-detectable phosphorylation of CREB in a time-dependent fashion (Figure 6). Phosphorylated CREB peaked at 10 min and plateaued till 30 min. (Figure 6A). Treatment of FT cells with membrane impermeable E2, E2-BSA, mimicked the effects of E2 and upregulated CREB phosphorylation (Figure 6B), suggesting involvement of membrane receptors.

**Figure 7:**
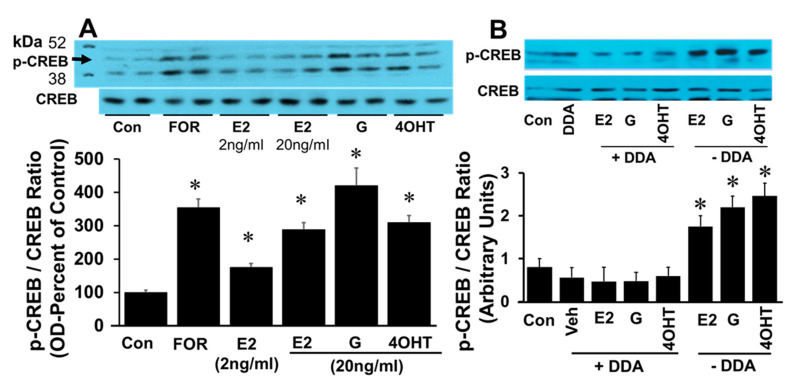
Panel (**A**) depicts a representative western blot and corresponding bar graph showing the changes in CREB phosphorylation in FT cells treated for 30 min with either forskolin (FOR; 50 μM), 17β-estradiol (E2; 2, 20 ng/mL), genistein (G; 20 ng/mL) or 4-OH-TCB (20 ng/mL). Panel (**B**) shows a representative western blot and corresponding bar graph depicting the modulatory effects of adenylyl cyclase inhibitor 2′,5′-dideoxyadenosine (DDA; 10 µM) on 17β-estradiol (E2; 20 ng/mL), genistein (G; 20 ng/mL) and 4OH-TCB (4OHT; 20 ng/mL) induced CREB phosphorylation in FT cells. Data represent (mean ± SEM) from separate experiments (*n* = 3). Results for phosphorylated CREB were normalized, with non-phosphorylated CREB and change in OD expressed as arbitrary units or percent of control. * *p* < 0.05 vs. untreated control, Con).

The effects of E2 on CREB phosphorylation were also mimicked by EEs. Treatment with 2 and 20 ng/mL E2 induced p-CREB in a concentration-dependent fashion. Exposure to forskolin, E2 (20 ng/mL), genistein (20 ng/mL), and 4-OH-TCB (20 ng/mL) increased CREB phosphorylation by 351 ± 2%, 275 ± 11%, 420 ± 65% and 300 ± 12%, respectively (Figure 7A), suggesting that EEs mimic the action of E2 action on CREB phosphorylation. To assess whether the phosphorylating effects of E2, genistein and 4OH-TCB involve adenylyl cyclase activation, the effects of these agents were tested in the presence of DDA (adenylyl cyclase inhibitor). The ability of E2, genistein and 4OH-TCB to stimulated phosphorylation of CREB was abrogated in the presence of DDA (Figure 7B).

**Figure 8:**
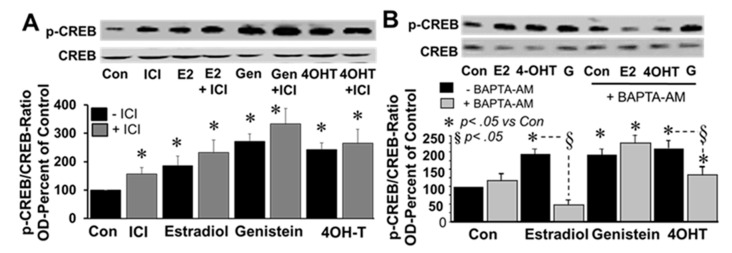
Panel (**A**) depicts, a representative western blot and corresponding bar graph showing the modulatory effects of the ERα/β antagonist ICI182780 (ICI; 1 µM) on 17β-estradiol (E2; 20 ng/mL), genistein (Gen; 20 ng/mL) and 4OH-TCB (4OHT; 20 ng/mL) induced CREB phosphorylation after treatment for 30 min. Panel (**B**) depicts a representative western blot and corresponding bar graph showing the modulatory actions of the intracellular Ca^2+^ chelator BAPTA-AM (1 µM) on 17β-estradiol (E2; 20 ng/mL), genistein (G; 20 ng/mL) and 4OH-TCB (4OHT; 20 ng/mL) induced CREB phosphorylation in FT cells incubated for 30 min. Data (mean ± SEM) represent the mean of three different experiments (*n* = 3). Results for phosphorylated CREB were normalized with non-phosphorylated CREB and expressed as percent of control. * *p* < 0.05 vs. untreated control, Con; ^§^ *p* < 0.05 vs. treatment without BAPTA-AM.

To investigate the involvement of ERα/β and intracellular calcium, the effects of E2, genistein and 4-OH-TCB were assessed in the presence and absence of ICI182780 (1 µM) and BAPTA-AM (cell permeable intracellular calcium chelator; 1 µM), respectively. Treatment of FT cells with ICI182780 induced CREB phosphorylation and was unable to block the stimulatory actions of E2, genistein or 4OH-TCB (Figure 8A). With regard to intracellular calcium, BAPTA-AM significantly inhibited the stimulatory effects of E2 and 4OH-TCB, but not the effects of genistein (Figure 8B).

**Figure 9:**
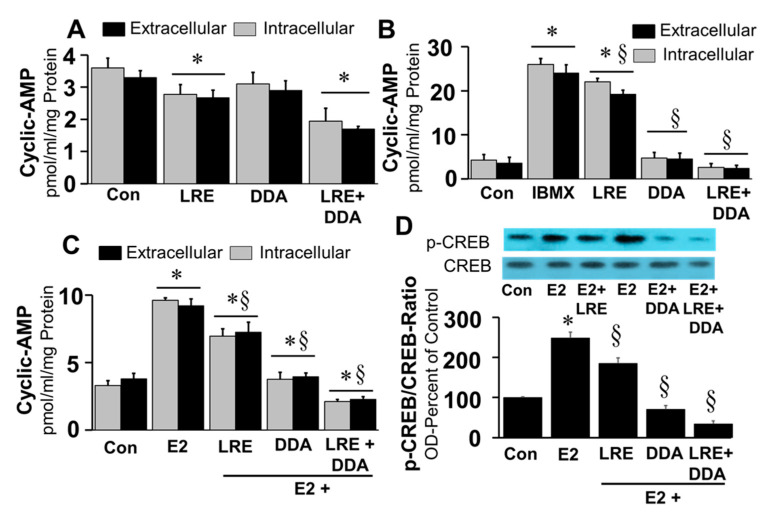
Figure depicting the inhibitory effects of sAC inhibitor, LRE1 (50 µM), tmAC inhibitor DDA (10 µM) and LRE1 (50 µM) plus DDA (10 µM) on basal (Panel (**A**)), IBMX (1 µM, Panel (**B**)); and E2 (20 ng/mL, Panel (**C**)), stimulated changes in intracellular and extracellular cyclic AMP levels in FT cells after treatment for 15 min. Panel (**D**) shows the effects of LRE1 (50 µM), DDA (10 µM) and LRE1 (50 µM) plus DDA (10 µM) on E2 (20 ng/mL) induced phosphorylation of CREB in FT cells treated for 30 min. Data for cAMP (mean ± SEM) represent the mean of three different experiments in triplicate (*n* = 3). Results for phosphorylated CREB were normalized with non-phosphorylated CREB and expressed as percent of control. * *p* < 0.05 vs. untreated control, Con; ^§^ *p* < 0.05 vs. treatment with E2, IBMX, LRE or DDA alone.

Calcium has diverse effects on transmembrane adenylyl cyclase (tmAC) activity whereas it activates soluble adenylyl cyclase (sAC) resulting in cellular cAMP generation. Our observation that, stimulated, but not basal, extracellular cAMP formation was inhibited by tmAC inhibitor DDA, and CREB phosphorylation by BAPTA-AM, a calcium chelator, suggests a potential role for both sAC and tmAC in driving cAMP formation in FT cells. Under basal conditions, treatment of FT cells with LRE1 (50 µM; sAC inhibitor) and DDA (10 μM) decreased intracellular and extracellular levels of cAMP by 19%–22% (*p* < 0.05) and 12%–14% (*p* > 0.5), respectively (Figure 9A). Moreover, the inhibitory effects were significantly greater (46%–48%) in cells treated with both LRE1 plus DDA. Treatment with LRE1, DDA or LRE1 plus DDA significantly inhibited IBMX and E2 induced changes in intracellular and extracellular cAMP (Figure 9B,C) in the following order of potency DDA plus LRE1 > DDA > LRE1. Moreover, treatment with LRE1, DDA or LRE1 plus DDA inhibited E2 induced CREB phosphorylation in the following order of potency being, LRE plus DDA > DDA > LRE1 (Figure 9D).

**Figure 10:**
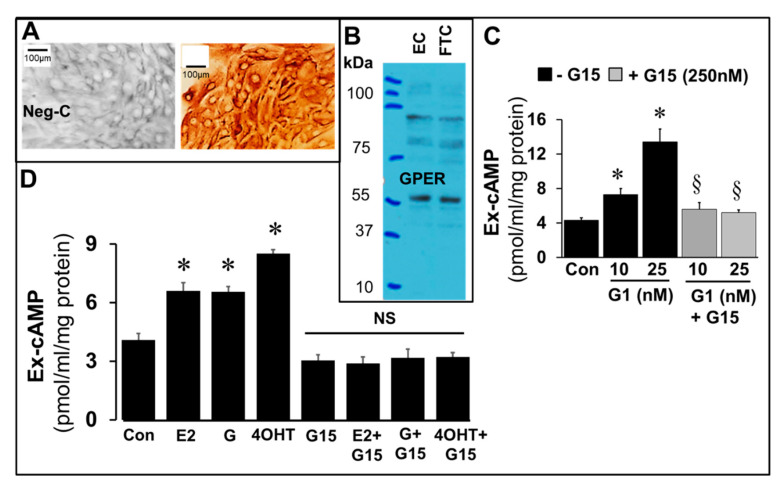
Panel (**A**) depicts representative photomicrographs (40× magnification) of bovine fallopian tube cells (FT cells; mixed cultures of epithelial cells and fibroblasts at 1:1 ratio). The photomicrograph on right depicts FT cells with positive staining for GPER whereas the left photomicrograph depicts the negative control (neg-C). Panel (**B**) shows a western blot for GPER (≈55 kDa) in FT cells (FTC) and vascular endothelial cells (EC). The bar graph in Panel (**C**) depicts the inhibitory effects of G15 (GPER antagonist; 250 nM) on extracellular (Ex) cAMP levels induced by G1 (25 nM) whereas Panel (**D**) shows the inhibitory effects of G15 (250 nM) on 17β-estradiol (E2; 20 ng/mL), genistein (G; 20 ng/mL) and 4OH-TCB (4OHT; 20 ng/mL). Data (mean and SEM) represent the mean of three different experiments (*n* = 3) in triplicates. Values were normalized to total protein concentration and cAMP level expressed as pmol/mL/mg protein. * *p* < 0.05 vs. control; ^§^ *p* < 0.05 vs. treatment with E2, IBMX, LRE or DDA alone.

The expression of GPER in FT cells was confirmed by immunostaining (Figure 10A) of cultured FT cells and western blotting in FT cell lysates (Figure 10B), and increase in cAMP production by FT cells incubated for 15 min with G1 (25 nM), a GPER agonist and its abrogation in presence of G15 (250 nM), a GPER antagonist (Figure 10C). Changes in cAMP formation in response to E2, genistein and 4OH-TCB were investigated in the presence and absence of G15 (250 nM), a GPER antagonist. Importantly, G15 abrogated the stimulatory effects of E2, genistein and 4OH-TCB on cAMP levels (Figure 10D).

## 5. Discussion

The interaction between FT epithelial cells and FT fibroblasts plays an important role in maintaining the biological and physiological function of the FT by generating multiple autocrine/paracrine factors [1,40]. Hence, we employed FT cells, a mixed culture of FT fibroblasts and FT epithelial cells (1:1 ratio), to assess the regulation of cAMP levels in the FT. Our finding that cAMP levels increased in the conditioned medium of FT cells in a time-dependent fashion suggests that the oviduct continuously synthesizes cAMP under basal conditions. The observation that basal levels of cAMP were significantly lowered in presence of sAC inhibitor LRE1 and tmAC inhibitor AC suggests their active role in cAMP synthesis by FT cells.

The current findings also indicate that, in the FT, a delicate balance exists between cAMP production and breakdown and that this balance defines cAMP levels in FT cells. Under basal conditions, we observed that only low levels of cAMP were achieved by FT cells; however, cAMP levels increased dramatically in the presence of adenylyl cyclase stimulators and PDE inhibitors (particularly the PDE-IV inhibitor Ro20-1724). The results with Ro20-1724 indicate that the PDE-IV isoform plays an outsized role in regulating cAMP levels in FT cells compared with PDE-I or PDE-III.

Our results show that E2, EEs, and phytoestrogens increase cAMP levels in FT cells by activating adenylyl cyclase and thereby stimulating production of cAMP. In support of this conclusion, we observed that the stimulatory actions of E2 on cAMP levels were mimicked by xenoestrogens (PCBs; TCBs, 4-OH-TCB and 4-OH-DCB) and a phytoestrogen (genistein) and were blocked by inhibition of adenylyl cyclase by DDA. The stimulatory effects of E2 and EE on cAMP production were accompanied by the expected increase in phosphorylated CREB, a downstream mediator of cAMP actions. In line with the conclusion that adenylyl cyclase mediates the effects of estrogens on cAMP levels, we found that the stimulatory effects of E2, EEs and a phytoestrogen on CREB phosphorylation were blocked by inhibition of adenylyl cyclase, again indicating a critical role for cAMP and adenylyl cyclase. The possibility that E2 increases cAMP by inhibiting PDE activity can be ruled out as it was unable to prevent cAMP degradation by FT cell homogenates (Supplementary Figure S5).

Most biological effects of E2 are mediated via nuclear receptors ERα and/or ERβ [41]. Our observation that ICI182789, an ER antagonist with equal affinity for ERα and ERβ [42], did not block the stimulatory effects of E2, EEs, or a phytoestrogen on cAMP production suggests that the effects of these agonist are not mediated by the classical nuclear receptors ERα or ERβ. Since the biologically active membrane estrogen receptor, referred to as GPER or GPER, is known to mediate rapid non-genomic actions of E2 [39,41], we investigated its role in E2-induced cAMP synthesis in FT cells. Similar to E2, equimolar concentrations of E2 tagged to BSA, which is membrane impermeable, induced cAMP synthesis. ICI182780 is known to be a GPER agonist [43]. The fact that ICI182780 stimulated cAMP production in FT cells suggests that the stimulatory actions of estrogens on cAMP production are mediated by GPER, and not by nuclear ERs. This contention is supported by our finding that FT cells expressed GPER and that the stimulatory effects of E2, EEs, and a phytoestrogen were blocked by G15, a GPER antagonist. The fact that E2-BSA increased cAMP levels suggests that E2 interacts with GPER on the plasma membrane and not the endoplasmic reticulum. However, it is possible that E2-BSA stimulates cAMP by indirect mechanism such as increasing intracellular calcium or engaging membrane-associated guanylate kinases or protein kinase A anchoring protein 5 which negatively inhibit cAMP production [44].

It appears that E2 and EEs, but not the phytoestrogen genistein, stimulate cAMP production in FT cells by a mechanism that requires intracellular calcium. This conclusion is based on our finding that chelation of intracellular calcium blocked the effects of E2 and EEs on cAMP production, yet the effects of genistein were not inhibited. Thus, the stimulatory mechanisms via which estrogens induce CREB phosphorylation can differ.

Our finding that EEs modulated local synthesis of cAMP and p-CREB within the FT suggests that EEs may play an important role in regulating the biology, physiology and development of the early embryo. In contrast to the cyclic effects of E2 on cAMP synthesis, continuous exposure to EEs could induce cAMP levels in a non-cyclic fashion that may induce deleterious effects on the reproductive system. Additionally, the deleterious actions may be further enhanced by PDE inhibitors widely used as anti-inflammatory drugs or in food products.

Treatment with E2 stimulated cAMP production in FT cells in a concentration-dependent manner; moreover, co-treatment with the adenylyl cyclase activator forskolin and/or isoproterenol enhanced the effects of E2 on cAMP production. This synergy suggests convergence of the signaling pathways for forskolin/E2 and isoproterenol/E2 at the level of adenylyl cyclase.

Our results suggest that cyclic changes in E2 levels, by inducing cyclic changes in cAMP production, may significantly influence the timing of physiology changes in the oviduct. Cyclic AMP is an important regulator of growth and muscle tone (relaxing agent), and dynamic changes in cAMP play an active role on oocyte maturation [5], fertilization, and embryo implantation, development, growth, and differentiation [21,45]. Exposure of embryos to the adenylyl cyclase stimulator forskolin or the PDE IV inhibitor rolipram prevented embryo development [24]. Notably, exposure of mice to caffeine, a PDE inhibitor at high concentrations, during the implantation window impaired embryo transport, embryonic development and uterine receptivity, leading to abnormal implantation and pregnancy loss [23]. These observations indicate that E2, together with other cytokines/hormones and PDEs, may regulate the physiological release of cAMP within the oviduct and influence the priming and development of the early embryo for implantation.

Similar to E2, EE’s bind to ERs and possess estrogenic properties. ER activation enables EE’s to act as endocrine disrupters and thereby induce pathological affects within the reproductive system [46]. Our findings provide the first evidence that EE’s stimulate cAMP synthesis in bovine FT cells. Genistein, a phytoestrogen, and the PCB analogs TCB, 4OH-DCB and 4-OH-TCB significantly induced cAMP synthesis in FT cells. Although they too bind to ERα and ERβ, they also bind to and activate GPER [43]. This suggests that GPER, the membrane estrogen receptor, may play a role in mediating the stimulatory effects of EEs on cAMP synthesis in FT cells. This contention is strongly supported by the observation that FT cells express GPER, cAMP levels are induced in FT cells treated with G1, a GPER agonist and the stimulatory actions of G1, E2 and EE were abrogated in presence of G15, a GPER antagonist.

Our findings suggest that EE’s may induce their deleterious effects in the reproductive system by modulating cAMP. Indeed, cAMP plays an important role in oocyte maturation, fertilization, early embryo development, implantation, growth and development [5]. Moreover, cAMP is a key regulator of muscle relaxation and facilitates successful progression of pregnancy until delivery [17]. cAMP is also a key regulator of immune function and induces anti-inflammatory actions by modulating local maternal immune responses and facilitating embryo implantation [13]. Importantly, conversion of extracellular cAMP to adenosine, a pro-fibrotic nucleoside in some tissues [47], may participate in tubal fibrosis by generating extracellular matrix deposition [48] thereby promoting fibrotic scar tissue formation that is implicated in tubal abnormalities associated with ectopic pregnancy [49]. Our findings suggest that EE’s can trigger non-cyclic cAMP production locally and potentially contribute to pathological remodeling of the fallopian tube that leads to adhesions.

Although we observed that E2 and EEs increased cAMP production, this was no guarantee that the measured increase was sufficient to activate downstream signaling. Since activation of PKA followed by phosphorylation of CREB is the major signaling pathway for cAMP, we measured, and confirmed, phosphorylation of CREB by E2 and EEs. Our finding that the stimulatory effects of E2 and 4OH-TCB, but not genistein, on CREB phosphorylation were abrogated by the intracellular calcium-chelator BAPTA-AM suggests that genistein-induced CREB phosphorylation is calcium independent and involves an alternative kinase. The fact that inhibition of adenylyl cyclase with DDA blocked the stimulatory effects of E2 and EEs on CREB phosphorylation suggests that CREB phosphorylation requires adenylyl cyclase activation. Since Ca^2+^ has been shown to activate adenylyl cyclase [50], cross talk between intracellular calcium signaling and adenylyl cyclase may contribute to the stimulatory effects of E2 and EEs on CREB phosphorylation. The fact that calcium signaling was involved in the effects of E2 and EEs further supports the conclusion that biologically important events occur when GPER receptors are stimulated by E2/EEs.

The major aim of this study was to determine the effects of E2 and EEs on cAMP production in FT cells. In line with this theme, there were two reasons for also examining the roles of PDE isoforms in FT cells. First, the impact on cAMP levels in FT cells of stimulating adenylyl cyclase with E2/EEs could be strongly dependent on the activity of PDEs within FT cells. Second, it is conceivable that E2/EEs increase cAMP levels not by activating AC, but rather by inhibiting PDEs. In the present study we observed that the stimulatory effects of E2 on cAMP levels are indeed importantly affected by the activity of PDEs (particularly PDE type 4). Furthermore, since E2 increased cAMP levels even when PDEs were inhibited, this indicates that the mechanism of action of E2 is not via inhibition of PDEs. Finally, because many PDE inhibitors are used clinically, the modulation of cAMP in FT cells may be of relevance for fertilization and early embryo development.

Our finding that cAMP levels were dramatically increased by the PDE-IV inhibitor Ro 20-1724 suggests that PDE-IV is the predominant PDE isoform in bovine FT cells. The fact that cAMP levels were very low under basal conditions and increased dramatically in response to PDE inhibitors suggests that low levels of cAMP in FT may facilitate early embryo priming and development and increases of cAMP during this period may induce deleterious actions. Indeed, exposure to PDE inhibitors induces deleterious actions on early embryo development, embryo transport, and uterine receptivity [22]. Since the effects of E2 are mimicked by EEs, their effects may also be enhanced in presence of PDE inhibiting agents.

In the FT, cAMP induces fluid production to facilitate transport [42], rhythmic contractility [50], and angiogenesis at the embryo–uterine interphase for placenta development [17,51]. In animal models, drugs/agents that increase cAMP levels by inhibiting its catabolism have been shown to impair embryo transport, embryonic development and implantation [22]. Moreover, high levels of estrogens in controlled ovarian hyperstimulation seems to have a negative impact on pregnancy rates. Herein, we observe that FT cells can produce large amounts of cAMP, and previously we found that in the oviduct cAMP can be sequentially converted to adenosine, which can generate cAMP via adenosine receptors [38]. We speculate, therefore, that abnormal increases in extracellular cAMP in response to EEs or PDE inhibitors may negatively influence early embryo development and priming for implantation. In-depth studies are required to further confirm this notion.

Recent studies suggest that cAMP formation may vary with various compartments. Activation of both tmAC and sAC results in intracellular cAMP formation. Activation of sAC plays a key role in phosphorylating nuclear CREB and is activated by calcium. Our observation that increases in intracellular cAMP levels and CREB phosphorylation in response to estradiol were abrogated by the soluble adenylyl cyclase inhibitor LRE1 and by BAPTA-AM suggests that this estrogen stimulates cAMP and pCREB by modulating intracellular calcium. The fact that E2-induced CREB phosphorylation and cAMP formation were also blocked by DDA, a tmAC inhibitor, suggests that increases in intracellular calcium by estradiol via tmAC activates sAC resulting in intracellular cAMP formation and CREB activation. The active role of sAC and tmAC in regulating cAMP synthesis in FT cells is also supported by our observation that LRE1 and DDA inhibited basal as well as IBMX induced cAMP levels. The relative role of sAC and tmAC in inducing cAMP synthesis in FT cells cannot be inferred from the current experiments; however, the fact that the inhibitory actions on cAMP formation were greatest in the presence of LRE1 plus DDA suggests that both tmAC and sAC are active within FT cells and that tmAC-mediated increases in intracellular calcium may activate sAC and this tmAC-sAC crosstalk may drive cAMP formation.

The pathophysiological significance of our finding that extracellular cAMP levels are induced by natural E2 and EE as much as by PDE inhibitors can only be speculated. The fact that various PDE inhibitors and cAMP inducers inhibit early embryo development [21,22,23,24,25,26,52] suggests that abnormal increases in cAMP may be deleterious. Indeed, cAMP has been shown to inhibit growth [7,8]; moreover, FT cells can convert cAMP to adenosine, which may result in activation of cAMP in distant cells, including the early embryo, via A_2B_ receptors [38]. Adenosine has been linked to fibrosis in some tissues, and fibrosis in FT is linked to ectopic pregnancy [47,48,49]. Thus, it is feasible that increased conversion of extracellular cAMP to adenosine may influence FT function and influence embryo transport. Interestingly, high levels of estrogens in controlled ovarian hyperstimulation seems to have a negative impact on pregnancy rates; however, whether this is associated with increases in extracellular cAMP remains to be confirmed.

Although our findings provide evidence that E2 and EEs induce cAMP in bovine FT cells, experiments in human cells are needed to confirm these actions. Another major limitation is that the work is in cell culture. Future studies should focus on the E2/GPER/AC/cAMP axis in vivo. Finally, the impact of high FT-derived cAMP on early embryo development should be confirmed using a co-culture model.

**Figure 11:**
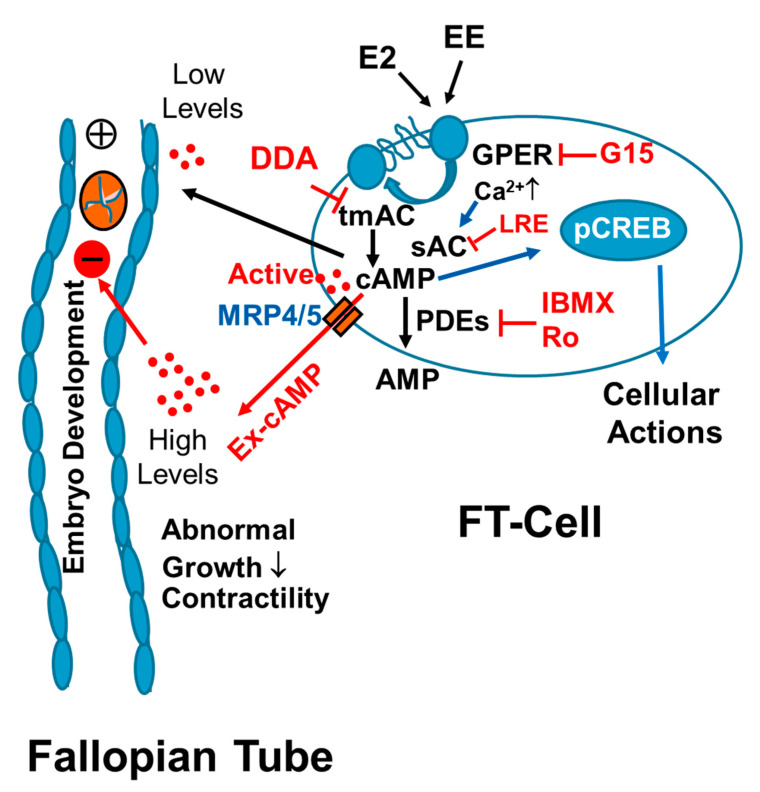
A schematic cartoon depicting the mechanism via which natural estrogens (E2) and environmental estrogens (EE) activate cyclic AMP (cAMP) generation by oviduct cells. Intracellular cAMP can mediate its cellular actions by phosphorylating CREB, and phosphodiesterases (PDEs) can metabolize/breakdown intracellular cAMP to AMP; moreover, cAMP is transported out of the cells by multidrug resistance-associated protein 4 and 5 (MRP4; MRP5). Using the G-protein coupled estrogen receptor (GPER) antagonist G15, transmembrane adenylyl cyclase (tmAC) inhibitor 2′5′-dideoxyadenosine (DDA) and soluble AC (sAC) inhibitor LRE1 (LRE), we demonstrate that in FT cells E2 and EE induce cAMP production via GPER/AC signaling. Since cAMP is a potent muscle relaxant and growth inhibitor, we hypothesize that increased generation of intracellular cAMP and extracellular cAMP (ex-cAMP) in a non-cyclic fashion may induce deleterious actions on early embryo development and transport through FT. Importantly the effects of both E2 and EE may be significantly enhanced in presence of PDE inhibitors such as the anti-inflammatory drugs rolipram (Ro) and IBMX or food products containing caffeine. Changes in cAMP levels may play an important role in early embryo development and its transport.

As summarized in Figure 11, our findings provide the first evidence that FT cells synthesize large amounts of cAMP and that cAMP is rapidly cleared by endogenous PDEs, in particular by PDE-IV. We also find that E2 and EE’s stimulate cAMP synthesis in FT cells via GPER/adenylyl cyclase signaling. Our findings suggest calcium mediated cross-talk between transmembrane adenylyl cyclase (tmAC) and soluble adenylyl cyclase (sAC) in regulating cAMP synthesis in FT cells. Hence, EE mediated non-physiologic stimulation of cAMP production, particularly in the presence of PDE inhibitors, may disrupt the progression of early embryo development and play an important role in reproductive pathologies. In conclusion, cAMP production within the FT may play a critical role during fertilization and early embryo development. Importantly, Exposure of the FT to high E2 levels (for example ovarian hyperstimulation in humans), EE’s and PDE inhibitors may result in abnormal non-cyclic induction of cAMP levels leading to deleterious effects on reproduction.

## Data Availability

All data supporting the findings of this study are available within the article and its supplemental data file or from the corresponding author upon reasonable request.

## Supplementary Materials

The following are available online at https://www.mdpi.com/article/10.3390/cells10051250/s1, Figure S1: representative photomicrograph of stained cultured fallopian tube fibroblasts plus epithelial cells, Figure S2: Cyclic AMP synthesis by fallopian tube epithelial cells and fibroblasts, Figure S3: Concentration-dependent modulatory effects of IBMX and mmIBMX on forskolin-induced cyclic AMP synthesis by fallopian tube cells, Figure S4: Modulation of intracellular and extracellular cyclic AMP levels by environmental estrogens in fallopian tube cells, Figure S5: Effects of estradiol on cyclic AMP degradation by fallopian tube cells.
