# Supplementary material for: Modulation of Cyclic AMP Levels in Fallopian Tube Cells by Natural and Environmental Estrogens
Source: Cells. 2021 May 19;10(5):1250. doi: 10.3390/cells10051250 (PMC8158772; doi:10.3390/cells10051250)
Supplement: Supplementary file 1 [file cells-10-01250-s001.zip › cells-1213647-supplementary.pdf]

Supplement

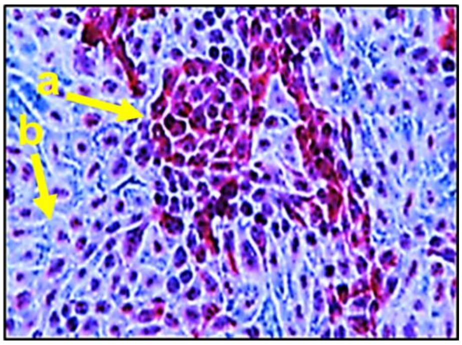

Figure S1: Figure depicts a representative photomicrograph (x100) of bovine fallopian tube cells (fallopian tube fibroblasts plus epithelial cells; primary cultures) with positive immunohistochemically labelling of fallopian fibroblasts with monoclonal antibodies against fibroblast vimentin (anti-vimentin VIM 3B4) marked with an arrow (a) and negatively stained epithelial cells (b). Reproduced from Cometti et al. Reproduction 2018, 155, 233-244, doi: 10.1530/REP-17-0425.

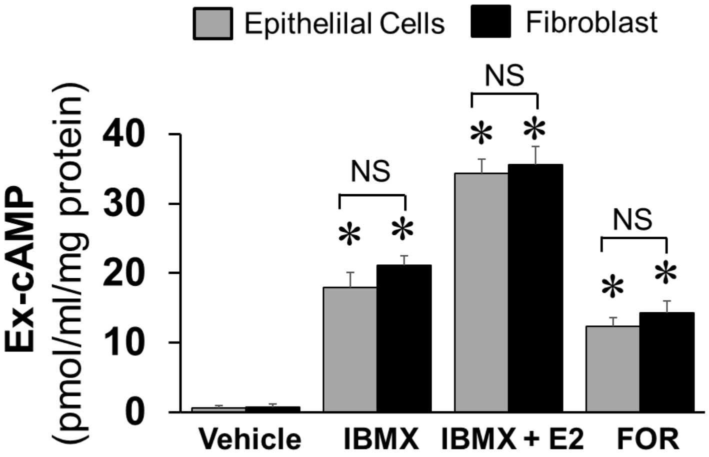

Figure S2: Cyclic AMP levels in fallopian tube epithelial cells and fibroblasts in presence and absence of the phosphodiesterase inhibitor IBMX, adenylyl cyclase activator forskolin (FOR) and IBMX plus 17β-estradiol (E2; 20 ng/ml). Cyclic AMP levels were measured in conditioned medium collected after 15 minutes in culture. The graph represents mean of three different experiments. Data (mean and SEM) are expressed as pmol/ml/mg protein.

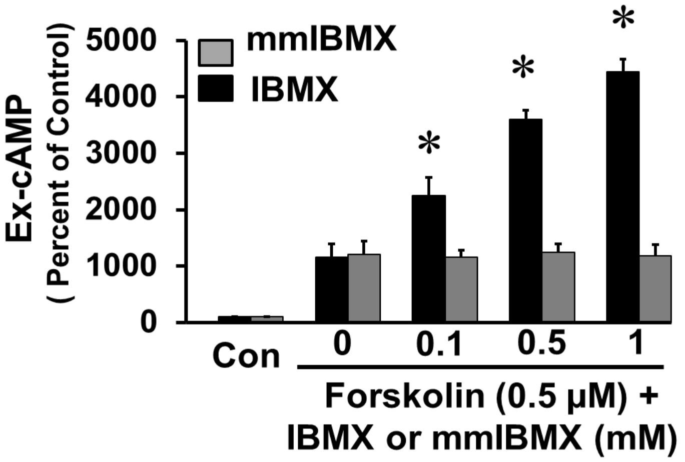

Figure S3: Concentration dependent modulatory effects of IBMX (0-1-1 mM) and mmIBMX (0.1-1 mM) on forskolin (0.5µM) induced extracellular (Ex) cAMP levels in fallopian tube epithelial cells and fibroblasts. Cyclic AMP levels were measured in conditioned medium collected after 15 minutes in culture. The graph represents mean of three different experiments. Data (mean and SEM) are expressed a percent change from untreated control (Con).

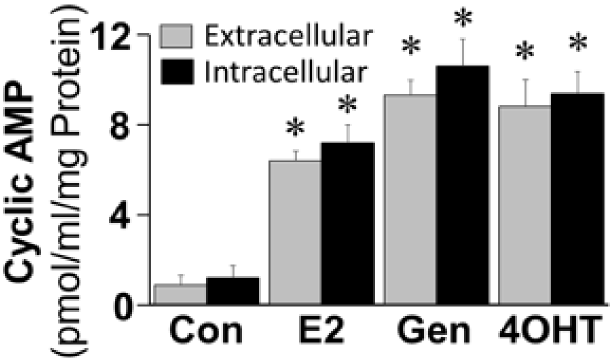

Figure S4: Bar graphs depicting the simultaneous changes in intracellular and extracellular cAMP in FT cells in response to 2ng/ml of E2, genistein (Gen) and 4OH-TCB (4OHT) by FTCs stimulated for 15 minutes. Data (mean ± SEM) represent the mean of three different experiments (n=3) in triplicates. Values were normalized to total protein concentration and the cAMP levels are expressed as pmol/ml/mg protein or percent of control. *p<0.05 vs respective control –E2.

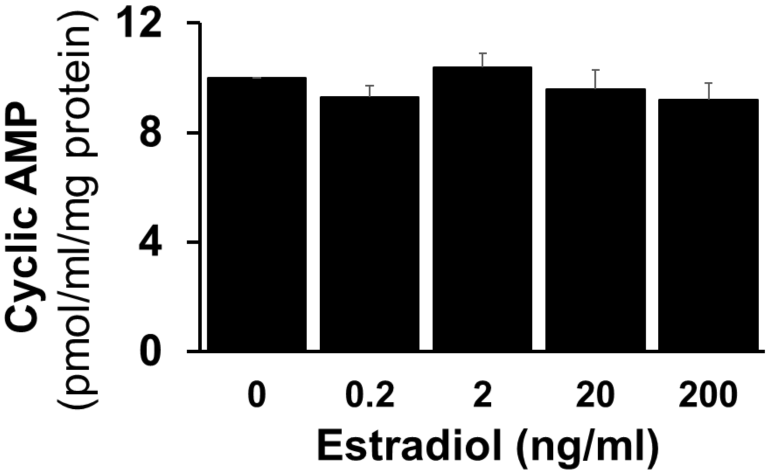

Figure S5: Bar graph showing the concentration dependent effects of 17β-estradiol on cyclic AMP degradation in mechanically disrupted FT cells after 4 hours of incubation with 10 µM cyclic AMP at 37°C. The levels of cyclic AMP in the conditioned medium did not significantly differ from samples incubated with our without E2. All values are SEM from 3 experiments, each in triplicate.
